# Macronutrients, vitamins and minerals intake and risk of esophageal squamous cell carcinoma: a case-control study in Iran

**DOI:** 10.1186/1475-2891-10-137

**Published:** 2011-12-20

**Authors:** Mahsa Jessri, Bahram Rashidkhani, Bahareh Hajizadeh, Maryam Jessri, Carolyn Gotay

**Affiliations:** 1 Human Nutrition Division, Department of Agricultural, Food and Nutritional Sciences, Edmonton Clinic Health Academy, University of Alberta, Edmonton, AB, Canada; 2 Alberta Institute of Human Nutrition, Edmonton Clinic Health Academy, University of Alberta, Edmonton, AB, Canada; 3 Community Nutrition Department, Faculty of Nutrition Sciences and Food Technology, Shahid Beheshti University of Medical Sciences, Tehran, Iran; 4 National Nutrition and Food Technology Research Institute (WHO Collaborating Center), Shahid Beheshti University of Medical Sciences, Tehran, Iran; 5 Department of Radiation Oncology, Kurdistan University of Medical Sciences, Kurdistan, Iran; 6 Oral Cancer Research Group, University of Queensland Center for Clinical Research, Brisbane, QLD, Australia; 7 Canadian Cancer Society Chair in Cancer Primary Prevention, School of Population and Public Health, Faculty of Medicine, University of British Columbia, Vancouver, BC, Canada

**Keywords:** Esophageal squamous cell carcinoma, macronutrients, vitamins, minerals, Iran

## Abstract

**Background:**

Although Iran is a high-risk region for esophageal squamous cell carcinoma (ESCC), dietary factors that may contribute to this high incidence have not been thoroughly studied. The aim of this study was to evaluate the effect of macronutrients, vitamins and minerals on the risk of ESCC.

**Methods:**

In this hospital-based case-control study, 47 cases with incident ESCC and 96 controls were interviewed and usual dietary intakes were collected using a validated food frequency questionnaire. Data were modeled through unconditional multiple logistic regression to estimate odds ratios (OR) and 95% confidence intervals (CI), controlling for age, sex, gastrointestinal reflux, body mass index, smoking history (status, intensity and duration), physical activity, and education.

**Results:**

ESCC cases consumed significantly more hot foods and beverages and fried and barbecued meals, compared to the controls (p < 0.05). After adjusting for potential confounders, the risk of ESCC increased significantly in the highest tertiles of saturated fat [OR:2.88,95%CI:1.15-3.08], cholesterol [OR:1.53, 95%CI: 1.41-4.13], discretionary calorie [OR:1.51, 95%CI: 1.06-3.84], sodium [OR:1.49,95%CI:1.12-2.89] and total fat intakes [OR:1.48, 95%CI:1.09-3.04]. In contrast, being in the highest tertile of carbohydrate, dietary fiber and (n-3) fatty acid intake reduced the ESCC risk by 78%, 71% and 68%, respectively. The most cancer-protective effect was observed for the combination of high folate and vitamin E intakes (OR: 0.02, 95%CI: 0.00-0.87; p < 0.001). Controls consumed 623.5 times higher selenium, 5.48 times as much β-carotene and 1.98 times as much α-tocopherol as the amount ESCC cases consumed.

**Conclusion:**

This study suggests that high intake of nutrients primarily found in plant-based foods is associated with a reduced esophageal cancer risk. Some nutrients such as folate, vitamin E and selenium might play major roles in the etiology of ESCC and their status may eventually be used as an epidemiological marker for esophageal cancer in Iran, and perhaps other high-risk regions.

## Background

Esophageal squamous cell carcinoma (ESCC) is the sixth most common cancer in the world and the fourth most common in the developing countries [1,2] with a remarkable variation in incidence in different regions of the world [1-3]. The latest epidemiologic report indicated the highest rate of ESCC to be in Iran, followed by other countries located on the "esophageal cancer belt" such as China, South Africa and France [2,4]. Both histologic types of esophageal malignancy (adenocarcinoma and squamous cell carcinoma) are highly lethal with five-year survival rates of less than 10% [5]. The incidence rate of esophageal cancer (EC) is 5-10 per 100,000 in North America and Europe, and more than 100 per 100,000 in China and Iran [3,6]. Although the incidence of EC is higher in males in most parts of the world [7], in very high incidence areas, such as Iran and China, the male to female ratio is close to one [7] and smoking and alcohol are not important risk factors as in western countries [8-18].

The alarmingly high incidence of ESCC and its equal sex distribution in Iran highlights the likelihood of a very strong environmental risk factor as the main culprit [19]. Gross nutritional deficiencies and unbalanced diets have long been suspected to play roles in ESCC risk, particularly in high-risk regions of the world where tobacco smoking and alcohol consumption are not very common [7,20]. Several studies have evaluated the effect of micronutrients, such as beta-carotene, folate, vitamin C and vitamin E on ESCC risk [21-30] and some have proved an inverse association [31-33]. In addition, dietary fat [33-35], butter, eggs [28], cholesterol [36] and starchy foods [28,37] have been directly related to the esophageal cancer risk, while dietary fiber is suggested to decrease the risk [33,34,38]. Data regarding the role of protein intake in ESCC etiology are conflicting [28,36,39].

While previous studies have mostly focused on food items in relation to ESCC risk, studying macro- and micronutrients could offer advantages mainly through providing better understanding of underlying mechanisms of disease [40]. According to Willet, when an association with overall intake of a nutrient is observed, the association with the etiology of a particular cancer type is strengthened, and hence by conducting analyses at the level of nutrients, maximal information on the cancer etiology will be obtained [41]. Previous studies in Iran have shown a widespread deficient intake of several nutrients such as riboflavin, vitamin A and vitamin C [42-44]; however, the impact of a wide range of macro- and micronutrient residual intakes in the etiology of ESCC has not been examined in this high-risk population. The aim of the present study was therefore, to evaluate the effect of major macronutrients, vitamins and minerals intakes on the risk of ESCC in a case-control study in Iran, and to compare the nutritional adequacy of cases and controls.

## Methods

### Population and sampling

This hospital-based case-control study was conducted in Kurdistan, a high-risk province of Iran. Cases were patients aged 40-75 years, who visited major general hospitals and had incident histologically-confirmed ESCC. Cases did not have history of carcinoma of other sites and were interviewed within 6 months after their ESCC was diagnosed. Controls were chosen from individuals admitted to the same hospitals as the cases for a wide spectrum of acute non-neoplastic diseases including traumas (25.9%, mostly fractures and sprains), surgical conditions (20.1%, mostly abdominal such as acute appendicitis and kidney stones), non-traumatic orthopedic conditions (4.2%, mostly disk disorders and back pain) and miscellaneous diseases (49.8%, including acute eye, nose, skin and throat disorders). Cases and controls were frequency-matched according to the sex and age (5-year groups).

### Measurements

Generally, 50 patients with ESCC and 100 hospital controls were interviewed face-to-face by professionally-trained interviewers using structured pre-tested questionnaires which evaluated socio-demographic characteristics (age, sex, education, monthly family income, and place of residence), smoking history (status, duration and intensity), eating habits (food and beverage temperature, cooking method), medical history, medication use (specifically aspirin and non-aspirin non-steroidal anti-inflammatory drugs (NSAIDs)), gastroesophageal reflux disease (GERD) symptoms (heartburn and acid regurgitation) and familial cancer history [45]. Questions on opium and alcohol consumption were not answered by our participants due to their cultural barriers and religious beliefs, and were hence excluded from the analyses. No proxy interviews were required.

Weight was measured with subjects clothed minimally, standing on digital scales (Soehne, Berlin, Germany) without shoes and was recorded to the nearest 100 grams. Height was measured using a non-stretch tape meter fixed to a wall with subjects standing without shoes and was recorded to the nearest 0.5 cm. Body mass index (BMI) was then calculated by dividing weight in kilograms by square of height in meters. Physical activity was measured using a validated questionnaire comprising of different metabolic equivalent (MET) categories [46], and it was then expressed as MET-hrs/day to estimate the physical activity level of participants [47,48].

Two patients were excluded from the analyses since their reported energy intakes were below or above 3 standard deviations from the mean, indicating errors in reporting dietary intakes [49]. We further excluded 5 patients due to missing information or due to poor response to dietary questions. Finally data analyses were conducted on 47 ESCC cases and 96 controls for whom the association of dietary patterns and food group intakes with ESCC risk had been documented previously [45,50].

### Dietary intake assessment

A validated semi-quantitative food frequency questionnaire (FFQ) was used by trained dietitians in face-to-face interviews to evaluate the usual dietary intakes of participants during the previous year [45,50]. Habitual dietary intakes of the controls 1 year before interview and the cases one year before diagnosis of ESCC were evaluated. The FFQ consisted of 125 Iranian food items and has previously shown to be a valid and reproducible tool for assessing food and nutrient intakes in Iranian adults [51,52]. Previous studies have revealed good correlations between dietary intakes assessed by this FFQ and those obtained from 24-h dietary recalls [52]. A comparison of crude, energy-adjusted and deattenuated correlation coefficients for overall nutrient intakes between 24-h dietary recalls and this FFQ have been 0.44 and 0.37 in ≤35 and >35 year-olds, respectively, and for individual nutrients it ranged from 0.24 to 0.71 in men and from 0.11 to 0.60 in women. On the other hand, the mean reliability coefficients, ranged from 0.48 in ≤35 year-olds to 0.65 in >35 year-olds. This FFQ produced exact agreement rates ranging from 39.6% to 68.3% in men and from 39.6% to 59.1% in women, respectively. The validity coefficients, with the sample correlation between the questionnaires and biological markers as the lower limit and the estimates from the triad method as the upper limit were 0.21-0.56 for protein and 0.37-0.61 for energy [52].

Portion sizes of consumed foods were specified according to the US Department of Agriculture (USDA) standard portion sizes (e.g. apple, 1 medium; bread, 1 slice; dairy, 1 cup) and were then converted to grams. When using the USDA portion sizes was impossible, household measures (e.g. beans, 1 tablespoon; chicken meat, 1 leg or wing; rice, 1 large or small plate) were used alternatively [53]. Patients were asked to report their consumption frequency on a daily, weekly or monthly basis, and data were then converted to the mean daily intakes assuming one month equals 30.5 days.

Average daily intake of each food item was calculated by multiplying the consumption frequency of each food by its standard item-specific portion size from the exchange list; these scores were then summed to estimate nutrient intakes. The estimates of nutrient intakes in the present paper are derived from the dietary sources alone. Since Iranian food composition table (FCT) is incomplete and provides data only on a few nutrients [54], analyses of energy and nutrients were carried out using the USDA FCT [55]. However, for some dairy products (such as Kashk), vetch, wild plum, mint, sweet canned cherry and sour cherry that are not listed in the USDA FCT, Iranian FCT was used alternatively [54]. For analyzing the energy and nutrient contents of mixed food items (e.g. pizza), usual restaurant recipes were used.

### Statistical analysis

For ordinal variables, chi-square test or Fisher's exact test and for continuous variables, Kruskal-Wallis test or t-student test were used to compare case and control groups. Macro- and micronutrient intakes were adjusted for total energy consumption using the residual method as suggested by Willet and Stampfer [56]. Energy-adjusted nutrients were then categorized into tertiles, due to the appropriateness of tertiles over quartiles for smaller sample sizes in case-control studies [57]. Tertile 1 served as the reference category for all regression analyses. Unconditional multiple logistic regression was used to estimate the odds ratio (OR) and 95% confidence intervals (CI) for the risk of ESCC tumor in the highest nutrient intake category compared to the lowest.

For comparison purposes, we calculated a base regression model and a fully-adjusted model for each analysis. The base model was adjusted for the matching variables, i.e. age (years) and sex (male/female), which are controlled for automatically by design. The fully-adjusted model, on the other hand, included the following covariates: age (years), sex (male/female), GERD symptoms (yes/no), BMI (≤24.9, >24.9 kg/m^2^kg/m^2^), smoking status (never/former/current), smoking intensity and duration (<20, ≥20 pack-years), physical activity (MET) (light/heavy), and education level (illiterate, literate).

Potential confounders were included in the multivariate models based on the review of literature, comparison of cases and controls and whether they modified the risk estimates 10% or more. These factors were selected since they are potentially related to both the disease outcome (ESCC risk) and also the risk factor (nutrient intakes). Other potential confounders such as monthly family income, place of residence (rural/urban), familial cancer history, cooking method and food and beverage temperature did not alter the main effect estimates for dietary factors and therefore were not included in the final models.

As a basis for trend testing, scores were constructed from the categorized variables as successive integers and were used in further analyses. All statistical tests were carried out using the Statistical Package for Social Sciences, version 16 (SPSS, Inc., Chicago, IL, USA) and a two-tailed P-value of <0.05 was considered statistically significant

### Ethical considerations

The present study was approved by all regional ethics committees in Iran and also the "Ethics Committee of the National Nutrition and Food Technology Research Institute", Shahid Beheshti University of Medical Sciences, Tehran, Iran. Written informed consents were taken from each subject prior to the interviews.

## Results

Table 1 presents the characteristics of 47 cases and 96 controls by gender categories. By design, age and sex distributions were similar among cases and controls. The majority of participants, particularly women, had no or limited education (p < 0.001).Despite the significant gender differences (p < 0.001), smoking status, duration and intensity did not differ among cases and controls (p > 0.05). Female cases experienced symptoms of GERD significantly more than their control peers (34.5% vs. 5.2%; p < 0.001). Among male participants, 11.1% of ESCC cases were overweight and obese (BMI > 24.9), compared to 47.4% in the control group (p = 0.04). In addition, BMI values and physical activity level of male and females differed significantly, with females being less physically active and more overweight/obese (p = 0.01). Compared to the controls, cases were more likely to consume hot foods and beverages (83.3% vs. 15.8%, in males and 58.6% vs. 15.5%, in females; p < 0.001) and fried/barbecued meals (44.4% vs. 7.9%, in males; and 20.7% vs. 5.2%, in females; p = 0.01).

**Table 1:** Distribution of cases and controls stratified by sex among selected risk factors in a case-control study of esophageal squamous cell carcinoma in Iran^1^

Variable	Male		Female		**P-value**^2^
	**Cases**	**Control**	**Cases**	**Control**	
			
Participants, *n*	18 (32.1)	38 (67.9)	29(33.3)	58(66.7)	
Age^3^, *year*	60.0(54.75-67.00)	60.0 (54.25-67.00)	57.0(50.00-70.50)	58.0 (48.75-72.25)	0.17
≤58	8(44.4)	17(44.7)	17(58.6)	32(55.2)	
>58	10(55.6)	21(55.3)	12(41.4)	26(44.8)	
Education, *year*					
Illiterate	14(77.8)	25(65.8)	28(96.6)	55(94.8)	<0.001
Literate	4(22.2)	13(34.2)	1(3.4)	3(5.2)	
Monthly family income, *US $*					
<300	15 (83.3)	33 (86.8)	28 (96.6)	53 (91.4)	0.14
≥300	3 (16.7)	5(13.2)	1 (3.4)	5 (8.6)	
Place of residence					
Rural	11(61.1)	20(52.6)	22(75.9)	28(48.3)*	0.80
Urban	7(38.9)	18(47.4)	7(24.1)	30(51.7)*	
Smoking history					
Never smoker	5(27.8)	17(44.7)	23(79.3)	51(87.9)	<0.001
Ex-smoker, pack year < 10	3(16.7)	3(7.9)	3(10.3)	4(6.9)	
Ex-smoker, pack year≥10	1(5.5)	11(28.9)	3(10.3)	1(1.7)	
Current smoker, pack year < 20	3(16.7)	4(10.5)	0(0.0)	1(1.7)	
Current smoker, pack year ≥20	6(33.3)	3(7.9)	0(0.0)	1(1.7)	
Having symptoms of GERD^4^	6(33.3)	6(15.8)	10(34.5)	3(5.2)*	0.31
Familial cancer history	1(5.6)	0(0.0)	3(10.3)	0(0.0)*	0.48
Physical activity					
Light	13(72.2)	21(55.3)*	16(75.9)	35(60.4)*	0.001
Heavy	5(27.8)	17(44.7)*	7(24.1)	23(39.6)*	
BMI, *kg/m*^*2*^	19.9(3.1)^5^	24.8(4.0)*	20.8(3.3)	25.7(4.3)*	0.01
≤24.9	16(88.9)	20(52.6)*	24(82.8)	27(46.6)	
>24.9	2(11.1)	18(47.4)*	5(17.2)	31(53.4)	
NSAIDs use					
Aspirin	2(11.2)	0(0.0)	1(5.6)	0(0.0)	0.63
Non-aspirin	3(7.9)	0(0.0)	1(3.4)	0(0.0)	
Food and beverage temperature					
Hot	15(83.3)	6(15.8)*	17(58.6)	9(15.5)*	0.03
Warm/cold	3(16.7)	32(84.2)*	12(41.4)	49(84.5)*	
Cooking method					
Fried/barbecued	8(44.4)	3(7.9)*	6(20.7)	3(5.2)*	0.14
Boiled	7(38.9)	21(55.3)*	10(34.5)	49(84.5)*	
Both	3(16.7)	14(36.8)*	13(44.8)	6(10.3)*	

GERD: Gastro-esophageal reflux disease; BMI: Body mass index; NSAIDs: Non-steroidal anti-inflammatory drugs

*Statistically significant between case and control groups (p < 0.05)

^1^Values are n (%), unless otherwise noted

^2^P-values were estimated using chi-square statistics, Fishers' exact test or independent t-test for the difference between genders

^3^Median (interquartile range (IQR))

^4^Patients who present with the typical GERD symptoms of heartburn and acid regurgitation

^5^Mean (SD)

The calorie-adjusted mean values for selected macronutrients and relative risk estimates of ESCC by tertiles of macronutrient intake residuals are presented in Table 2 and Additional file 1. Cases consumed significantly more SFA and discretionary calories (energy derived from solid fat and added sugar), compared to the controls (p = 0.006). On the other hand, controls consumed significantly more (n-3) fatty acids, dietary fiber, carbohydrate and vegetable oil than their case peers (p = 0.04).

**Table 2:** Relationship between energy-adjusted macronutrient intakes and risk of esophageal squamous cell carcinoma in a case-control study in Iran

**Macronutrients**	**Tertiles of Intake**^**1 **^**OR (95%CI)**							
	
	**Model 1**^**2**^				**Model 2**^**3**^			
				
	**Tertile1**^**5**^	**Tertile2**	**Tertile3**	**P-trend**^**6**^	**Tertile 1**^**4**^	**Tertile 2**	**Tertile 3**	**P-trend**^**5**^
Total energy, *Kcal Number*	1.00 (46)	0.80 (0.24-2.13) (48)	1.11 (0.25-1.98) (49)	0.52	1.00	0.62 (0.03-2.65)	1.23 (0.86-2.14)	0.29
Total fat, *g Number*	1.00 (46)	1.23 (1.04-2.95) (46)	1.94(1.05-3.28) (51)	0.02	1.00	1.11 (0.80-2.67)	1.48 (1.09-3.04)	0.005
SFA, *g Number*	1.00 (48)	1.70 (1.21-4.93) (47)	3.52 (1.10-3.89) (48)	0.01	1.00	1.32 (1.20-2.93)	2.88 (1.15-3.08)	0.01
PUFA, *g Number*	1.00 (47)	2.83 (0.34-3.60) (48)	0.71 (0.13-1.62) (48)	0.98	1.00	1.19 (0.42-2.57)	0.98 (0.31-2.64)	0.14
MUFA, *g Number*	1.00 (48)	1.39 (0.95 -2.79) (47)	1.39 (0.29-2.16) (48)	0.23	1.00	0.97 (0.15-1.74)	1.19 (0.42-2.75)	0.81
(n-3)fatty acids, *g Number*	1.00 (48)	0.42 (0.21-0.75) (47)	0.51 (0.08-0.90) (48)	0.01	1.00	0.86 (0.16-0.97)	0.32 (0.07-0.84)	<0.001
Dietary fiber, *g Number*	1.00 (48)	0.72 (0.31-2.18) (47)	0.46 (0.01-0.88) (48)	<0.001	1.00	0.71 (0.02-2.03)	0.29 (0.13-0.76)	0.02
Carbohydrate, *g Number*	1.00 (46)	0.70 (0.31-2.93) (46)	0.17 (0.06-0.92) (51)	0.04	1.00	0.79 (0.32-1.56)	0.22 (0.05-0.84)	<0.001
Protein, *g Number*	1.00 (48)	1.25 (0.59-2.74) (48)	1.61 (1.49-4.13) (47)	<0.02	1.00	1.13 (0.54-1.64)	1.93 (0.60-3.18)	0.52
Cholesterol, *mg Number*	1.00 (47)	0.92 (0.09-1.39) (49)	3.71(1.49-2.60) (47)	<0.001	1.00	0.68 (0.22-1.73)	1.53 (1.41-4.13)	<0.001
Vegetable Oil, *g*^*6 *^*Number*	1.00 (47)	0.59 (0.21-3.58) (47)	0.95 (0.16-2.24) (49)	0.87	1.00	1.25 (0.35-1.93)	1.44 (0.08-2.23)	0.61
Discretionary calorie, *% total energy intake*^7 ^*Number*	1.00 (48)	1.68 (1.07-3.95) (48)	2.33(1.58-2.90) (47)	<0.001	1.00	1.17 (1.02-2.65)	1.51 (1.06-3.84)	0.002

OR: Odds ratio; CI: Confidence interval; SFA: Saturated fatty acid; PUFA: Poly unsaturated fatty acid; MUFA: Mono unsaturated fatty acid

^1^Nutrient intakes are adjusted for energy intake using the residual method [56]

^2^Base model; adjusted for age (years) and sex (male/female)

^3^Fully-adjusted model; adjusted for age (years), sex (male/female), gastroesophageal reflux disease symptoms (yes/no), body mass index (≤24.9, >24.9 kg/m^2^), smoking status (never/former/current), smoking intensity and duration (<20, ≥20 pack-years), physical activity (MET) (light/heavy), and education level (illiterate, literate)

^4^Reference category

^5^The P-value for trend was calculated using the linear regression coefficient for the tertiles of macronutrient intake

^6^Described as fat from vegetable oil, fish, nuts and seeds sources

^7^Described as the energy derived from solid fat and added sugar

In the fully-adjusted model, those in the highest tertile of SFA intake had 2.88 times higher ESCC risk (95% CI: 1.15-3.08; p-trend = 0.01), followed by those in the highest intake tertile of cholesterol (OR: 1.53, 95% CI: 1.41-4.13; p-trend < 0.001), discretionary calorie (OR: 1.51, 95% CI: 1.06-3.84; p-trend = 0.002) and total fat intake (OR: 1.48, 95% CI: 1.09-3.04; p- trend = 0.005). On the other hand, being in the highest tertiles of carbohydrate, dietary fiber and (n-3) fatty acid reduced the risk of ESCC by 78%, 71% and 68%, respectively. In the preliminary age- and sex- adjusted analysis (original matching criteria), a positive association also emerged with an increased protein intake, which was not significant in the fully-adjusted model.

The adjusted mean intakes of vitamin A, β-Carotene, vitamin D, vitamin E, α-Tocopherol, thiamin, riboflavin, vitamin B_6_, folate, vitamin B_12_, vitamin C, iron, calcium, phosphorus, methionine and selenium were significantly higher among controls compared to ESCC cases (p < 0.05), while average adjusted sodium intake was significantly higher among cases compared to the controls ( p < 0.001) (Additional file 2). Controls consumed 623.5 times as much selenium (p < 0.001), 5.48 times as much β-carotene and 1.98 times as much α-tocopherol as the amount ESCC cases consumed. In the fully-adjusted model, the most protective effects against ESCC risk were associated with higher intakes of folate (OR: 0.08, 95% CI: 0.02-0.90; p-trend <0.001) and vitamin E intakes (OR: 0.11, 95% CI: 0.03-0.74; p-trend < 0.001), closely followed by selenium (OR: 0.15, 95% CI: 0.01-0.76; p-trend < 0.001), vitamin B_6 _(OR: 0.17, 95%CI: 0.05-0.91, p-trend = 0.003) and riboflavin intakes (OR: 0.22, 95%CI: 0.07-0.86; p-trend = 0.01) (Table 3). Being in the highest tertile of sodium intake residual was associated with 1.49 fold increase in the ESCC risk (p < 0.001). A significant inverse relationship between ESCC risk and higher intakes of α-tocopherol, thiamine and potassium observed in the base model, disappeared when other potential confounders were taken into account.

**Table 3:** Relationship between energy-adjusted micronutrients intakes and risk of esophageal squamous cell carcinoma in a case-control study in Iran^1^

**Micronutrients**	**Tertiles of Intake**^**2 **^**OR (95%CI)**							
	
	**Model 1**^**3**^				**Model 2**^**4**^			
				
	**Tertile1**^**6**^	**Tertile2**	**Tertile3**	**P-trend**^**7**^	**Tertile 1**^**5**^	**Tertile 2**	**Tertile 3**	**P-trend**^**6**^
Vitamin A, *RAE Number*	1.00 (48)	0.82 (0.19-1.50) (49)	0.93 (0.50-2.86) (46)	0.94	1.00	0.83 (0.09-1.86)	0.72 (0.38-2.12)	0.53
β-carotene, *μg Number*	1.00 (48)	1.59 (0.32-2.86) (47)	1.29 (0.51-3.65) (48)	0.71	1.00	1.21 (0.13-2.68)	1.07 (0.81-2.13)	0.14
Vitamin D, *μg Number*	1.00 (48)	1.10 (0.62-1.75) (47)	0.28 (0.17-0.89) (48)	<0.001	1.00	0.84 (0.39-2.74)	0.28 (0.02-0.91)	<0.001
Vitamin E, *mg TE Number*	1.00 (47)	0.19 (0.03-0.94) (48)	0.07 (0.01-0.63) (48)	<0.001	1.00	0.32 (0.12-0.91)	0.11 (0.03-0.74)	<0.001
α-tocopherol, *mg Number*	1.00 (47)	0.61 (0.12-0.95) (48)	0.26 (0.09-0.74) (48)	<0.001	1.00	0.86 (0.14-1.17)	0.47 (0.02-1.85)	0.31
Thiamine, *mg Number*	1.00 (47)	0.57(0.21-2.73) (48)	0.41 (0.05-0.89) (48)	0.04	1.00	0.85 (0.61-1.58)	0.34 (0.06-2.85)	0.97
Riboflavin, *mg Number*	1.00 (47)	0.90 (0.22-1.85) (48)	0.33 (0.15-0.87) (48)	<0.001	1.00	1.90 (0.17-2.12)	0.22 (0.07-0.86)	0.01
Niacin, *mg Number*	1.00 (48)	0.37 (0.06-2.10) (47)	0.48 (0.10-1.69) (48)	0.17	1.00	0.86 (0.05-2.63)	0.38 (0.15-1.82)	0.09
Panthothenic acid, *mg Number*	1.00 (47)	0.55 (0.07-2.16) (48)	0.86 (0.29-1.11) (48)	0.50	1.00	0.86 (0.20-1.18)	0.49 (0.35-2.08)	0.74
Vitamin B6, *mg Number*	1.00 (47)	0.48 (0.15-0.79) (47)	0.11(0.08-0.93) (49)	<0.001	1.00	0.76 (0.12-2.33)	0.17 (0.05-0.91)	0.003
Folate, *μg Number*	1.00 (48)	0.26 (0.07-0.90) (47)	0.08 (0.01-0.92) (48)	<0.001	1.00	0.32 (0.01-0.57)	0.08 (0.02-0.90)	<0.001
Vitamin B_12_, *μg Number*	1.00 (47)	0.58(0.19-1.83) (49)	1.02 (0.39-2.12) (47)	0.14	1.00	0.87 (0.10-2.61)	1.33 (0.60-3.03)	0.15
Vitamin C, *mg Number*	1.00 (47)	0.69 (0.13-0.75) (49)	0.39 (0.11-0.84) (48)	0.02	1.00	0.76 (0.09-2.43)	0.37 (0.11-0.93)	0.02
Iron, *mg Number*	1.00 (47)	0.51 (0.07-0.93) (49)	0.69 (0.17-2.60) (47)	0.66	1.00	0.72 (0.35-1.63)	0.61 (0.22-2.48)	0.13
Calcium, *mg Number*	1.00 (47)	0.27(0.12-0.87) (49)	0.17 (0.03-0.94) (47)	<0.001	1.00	0.51 (0.17-1.82)	0.49 (0.15-0.87)	0.03
Phosphorous, *mg Number*	1.00 (48)	1.09 (0.62-3.29) (47)	0.77 (0.01-2.56) (48)	0.62	1.00	1.35 (0.11-2.95)	1.31 (0.36-2.60)	0.92
Potassium, *mg Number*	1.00 (48)	0.51 (0.07-1.98) (47)	0.24 (0.11-0.78) (48)	0.03	1.00	0.51 (0.18-2.93)	0.23 (0.03-1.76)	0.44
Sodium, *mg Number*	1.00 (48)	1.13 (0.22-2.07) (47)	1.52 (1.17-3.44) (48)	<0.001	1.00	1.17 (1.05-2.15)	1.49 (1.12-2.89)	<0.001
Zinc, *mg Number*	1.00 (47)	0.79 (0.16-0.73) (48)	0.49 (0.11-0.85) (48)	0.01	1.00	1.39 (0.58-2.17)	0.73 (0.12-0.98)	0.01
Methionine, *g Number*	1.00 (47)	0.85 (0.09-2.34) (48)	0.63 (0.09-0.96) (48)	<0.001	1.00	0.79 (0.06-1.98)	0.29 (0.13-0.95)	0.004
Selenium, *μg Number*	1.00 (47)	0.32 (0.12-0.94) (48)	0.12 (0.04-0.69) (48)	0.03	1.00	0.63 (0.12-0.91)	0.15 (0.01-0.76)	<0.001

OR: Odds ratio; CI: Confidence interval; RAE = Retinol Activity Equivalents; TE= Tocopherol Equivalents

^1^Only micronutrients from food sources are considered.

^2^Nutrient intakes are adjusted for energy intake using the residual method [56]

^3^Base model; adjusted for age (years) and sex (male/female)

^4^Fully-adjusted model; adjusted for age (years), sex (male/female), gastroesophageal reflux disease symptoms (yes/no), body mass index (≤24.9, >24.9 kg/m^2^), smoking status (never/former/current), smoking intensity and duration (<20, ≥20 pack-years), physical activity (MET) (light/heavy), and education level (illiterate, literate).

^5^Reference category

^6^The P-value for trend was calculated using the linear regression coefficient for the tertiles of micronutrient intake

Table 4 shows the OR (95% CI) for the joint effect of vitamin E and folate intake residuals on ESCC risk. After mutual adjustment for several potential confounders, the combination of high intakes of both chemicals was associated with a strong protective effect against ESCC risk (OR: 0.02, 95% CI: 0.00-0.87; p < 0.001).There was a statistically significant interaction between vitamin E and dietary folate intake when evaluated in the model (p-value for interaction = 0.03).

**Table 4:** Odds ratios (ORs) and 95% confidence intervals (CI) for joint effect of energy-adjusted vitamin E and folate intake on esophageal squamous cell carcinoma risk in a case-control study in Iran1,2

	Folate					
	
	Model 1^3^			Model 2^4^		
		
	Low	Medium	High	Low	Medium	High
Vitamin E						
Low *Number*	1.00 (32)	0.51 (0.19-0.72) (15)	0.44 (0.13-0.90) (4)	1.00	0.48 (0.11-0.75)	0.48 (0.11-0.75)
Medium *Number*	0.63 (0.09-0.86) (14)	0.08 (0.01-0.47) (23)	0.07 (0.01-0.69) (11)	0.52 (0.09-0.81)	0.05 (0.01-0.39)	0.05 (0.02-0.41)
High *Number*	0.22 (0.01-0.79) (2)	0.05 (0.00-0.76) (9)	0.01 (0.00-0.79) (33)	0.19 (0.05-0.66)	0.04 (0.01-0.42)	0.02 (0.00-0.87)

^1^Nutrient intakes are adjusted for energy intake using the residual method [62]

^2^P for interaction = 0.03

^3^Base model; adjusted for age (years) and sex (male/female)

^4^Fully-adjusted model; adjusted for age (years), sex (male/female), gastroesophageal reflux disease symptoms (yes/no), body mass index (≤24.9, >24.9 kg/m^2^), smoking status (never/former/current), smoking intensity and duration (<20, ≥20 pack-years), physical activity (MET) (light/heavy), and education level (illiterate, literate)

## Discussion

Results of the present study suggest that among macronutrients, consuming more carbohydrate, dietary fiber and (n-3) fatty acids and among micronutrients, higher intakes of folate, vitamin E and selenium have the most protective effects against ESCC in a high-risk population in Iran. An increased ESCC risk estimate was observed among those with highest intakes of SFA, cholesterol, discretionary calorie, sodium and total fat. Most importantly, being in the highest tertile of joint folate and vitamin E intake was associated with 98% reduction in the ESCC risk.

This is the first study in a high-risk population to evaluate the impact of a wide range of macronutrients, minerals and vitamins on risk of ESCC. Similar to previous case-control studies, we found a decreased ESCC risk associated with higher intakes of nutrients with plant origin and an increased risk for intake of several nutrients found primarily in animal-based foods [28,29,58-61]. In addition to the differences in nutritional composition of animal- and plant-based foods that contribute to this effect, heterocyclic amines that are potent mutagens, and animal carcinogens formed during cooking of meats are also responsible [62]. The highest level of mutagenic activity is produced during frying, broiling and barbecuing animal products [59], which could potentially injure the esophageal mucosa [63].

Epidemiological studies have shown a positive association between total fat, cholesterol and SFA intakes with ESCC and esophageal adenocarcinoma risk [33,39,57,64-67]. In the present study, more than one-thirds of total energy intake among ESCC cases was derived from dietary fat and those with higher intakes of total fat, SFA, cholesterol and discretionary calories had an increased risk of ESCC. According to the Dietary Guidelines for Americans 2005 [68], 12-20% of total energy intake could be taken from discretionary calories, while in the present study more than 50% of total calories consumed were from discretionary calories, which is of concern since those in higher tertiles of discretionary calorie intake had about 1.5 times higher risk of ESCC. Since all our analyses were adjusted for usual adult BMI, the risk-enhancing effect of high fat diet on ESCC observed in the present study was independent of adiposity, which is a strong risk factor for carcinogenesis. Further effect modification by BMI revealed that although ESCC risk was higher among those with higher BMI values, p for interaction was not significant (data not shown). However, this effect is likely to have been underestimated since ESCC patients tend to decrease their dietary fat intake in an effort to prevent exacerbation of reflux symptoms, and hence our result is likely to have been distorted through underestimation of magnitude of true association for dietary fat. Although some studies have shown an inverse association between EC risk and intakes of added oil and PUFAs [33], we failed to show a significant relationship.

Findings of previous studies have been inconclusive regarding the role of protein intake in esophageal cancer risk with some classic studies suggesting an inverse relationship [28,69]. We observed a positive association between protein intake and ESCC risk, which is in line with more recent studies [33,38,39,57,67,70]; however, this effect was only statistically significant in the age- and sex-adjusted model.

High intake of dietary fiber in the present study decreased ESCC risk by about 70%. Although few studies have questioned the role of dietary fiber in cancer protection [71-74], most have proved a strong inverse association between fiber intake and risk of ESCC, esophageal adenocarcinoma and stomach cancer [33,34,38,57,59,60,67,70,75,76]. The role of carbohydrate intake in esophageal cancer risk is not yet clear with some studies showing a negative association [57], but not all [77-79]. In the present study, participants with higher carbohydrate intakes had markedly reduced ESCC risk. However, carbohydrate intake was also negatively correlated with fat intakes (correlation coefficient = -0.615) and hence a higher percentage of carbohydrate may just reflect lower intakes of fat and explain its inverse association with ESCC [57]. In addition, higher consumption of carbohydrate could be reflection of more plant-based food intakes, and especially fruit and vegetable; although in the present research, the correlation between carbohydrate and fruits and vegetable intakes was not significant (r = 0.064).

It has been suggested that the cancer-protective effects of fruits and vegetables intake is mediated through their several antioxidants and dietary components, such as folate, vitamin A, β-carotene, vitamin C and dietary fiber [64,69,80-83]. Previously we showed an inverse association between fruit and vegetable consumption and risk of ESCC in the same population [50], and in the present study, after adjustment for fruit and vegetable intake, the association of dietary folate, vitamin E and selenium with ESCC risk remained significant, suggesting an independent protective role for these nutrients.

Epidemiological evidence regarding the role of folate intake in ESCC risk are scanty [21,67,84,85]. Findings of the present research are in agreement with those of the previous studies showing a strong inverse association between dietary folate intake and risk of ESCC [21,22,57,67,85]. However, it is likely that we have more clearly observed an inverse relationship between folate intake and ESCC compared to other studies [22], since in Iran there is no mandatory folate fortification and the use of dietary supplements is very uncommon; hence, folate is mainly taken from diet in this population. In populations with mandatory folate fortification and frequent supplement use, most of the population may have sufficient folate intakes to prevent cancer from these sources and little further benefit may be seen for dietary folate intake. On the other hand, the marked cancer-protective effect we observed for high folate intakes could be partly attributed to the comparatively high rates of folate intake deficiency, as more than 90% of cases and 50% of controls in this study had intakes below the Recommended Dietary Allowances (RDA) (data not shown) [86]. Folate is an important cofactor in DNA metabolism and its deficiency has been linked to higher risk of epithelial tumors [22,23,25,70,87]. Several mechanisms have been proposed to explain the protective effect of folate, which are mainly focused on prevention of hypomethylation and maintenance of the DNA repair system by influencing the nucleotide pool for DNA replication and repair [88-90].

Similar to folate, vitamin B_2_, B_6_, B_12 _and methionine have major roles in one-carbon metabolism. Previous studies in Iran have reported inadequate riboflavin intakes among patients with esophageal cancer [44]. This is in line with findings from this research and those of previous studies, which have shown protective effects for this nutrient against the risk of EC [28,69,82,91]. Inadequate vitamin B_6 _intakes among ESCC cases also, might leads to chromosome breakage [92], defective DNA synthesis and methylation and could increase the ESCC risk [35,67,85].

In this study, we failed to observe a significant association between vitamin B_12 _intake and ESCC risk. However, it has been suggested that the positive relationship between vitamin B_12 _and EC risk observed in some studies [67], could be explained by the fact that vitamin B_12 _is derived exclusively from animal sources and hence may be simply a marker for consumption of animal protein and other factors or nutrients in these foods [67]. It has also been documented that individuals living in the high ESCC risk regions have significantly lower vitamin B_9 _and B_12 _intakes, compared to those living in the low risk areas [93] and those with vitamin B_12 _deficiency disorders (e.g. pernicious anemia) are at greater risk of EC [94,95]. Overall, causal relationship between vitamin B_12 _intake and ESCC risk is not yet clear and evidence in this regard is lacking. In the present study, high methionine intake was inversely associated with ESCC risk, which might be explained through its involvement in SAM production, which is necessary for retaining folate in body [96]

Higher intakes of vitamin E and vitamin D in this study were associated with about 90% and 70% ESCC risk reduction, respectively. The finding of a strong inverse association between ESCC with vitamin D intake in this study is consistent with some studies [97,98], although in contrast with others [67]. This contradiction could be explained by the fact that dietary and supplemental vitamin D intakes only comprise a relatively small proportion of the variation in 25-hydroxy vitamin D levels in the body, and sunlight exposure, skin pigmentation, geographic region of residence, season, BMI, and differences in vitamin D receptor expressions (genetic differences) are the major predictors of 25(OH) D levels in the body [99,100].

Dietary antioxidants (vitamin C, β-carotene and vitamin E) have been shown to decrease the EC risk [27,28,33,38,39,65,82,98,101] through several mechanisms such as deactivating excited oxygen molecules and preventing lipid peroxidation. [27,28,38,61,65]. Dietary antioxidants also play major roles in prevention of damage to the mucosa of the upper aerodigestive tract caused by oxidative stress of smoking and alcohol consumption. In the Linxian China trial, supplementation with a combination of vitamin E, β-carotene, and selenium reduced the incidence of esophageal/gastric cardia cancer by 6% [102]; this is consistent with our findings of a strong inverse association between vitamin E and selenium with the risk of ESCC. Dietary selenium is inversely associated with cell cycle predictors of neoplastic progression and the ESCC risk [103-105]. The potential of combined supplementation in the Linxian trial in reducing the EC risk has been mainly attributed to the effect of selenium, which has a highly deficient intake in Chinese population. This is in agreement with results of our study in which none of the cases had adequate selenium intakes, and higher intake of selenium was associated with 85% reduction in ESCC risk.

Vitamin A and β-carotene were not significantly associated with ESCC risk in our population, which is in line with several studies [57,69,81,98,106] and in contrast with others [107,108]. Our inability to detect a significant relationship may be due to considering vitamin A intakes from both plant and animal origins together, while it has been suggested that vitamin A of plant origin is associated with decreased ESCC risk, whereas vitamin A of animal origin increases the level of risk [67,109]. In addition, the protective effect of high carotene intake observed in some of the previous studies could have been mediated through high intakes of plant-based foods, which contain different micronutrients and hence contribute to the general effect [59,61,98]. High intake of vitamin C in this study was associated with more than 60% reduction in ESCC risk. Vitamin C, as an important antioxidant and inhibitor of endogenous synthesis of N-nitroso compounds [109-111] prevents carcinogenesis of esophageal cells [110]. However, an intervention trial in China failed to show a reduction in esophageal cancer incidence and mortality in individuals taking 120 mg/day vitamin C for 5.25 years [102].

In the present study, higher sodium intake was associated with almost 50% increase in the ESCC risk. Some epidemiological studies have suggested a role for higher salt intake in carcinogenesis. Although salt is not a carcinogen per se, it acts as an irritant to the esophageal protective mucosal layer, which results in inflammatory regenerative response, increased DNA synthesis and cell proliferation [23] and may also enhance carcinogenesis induced by other carcinogens [112].

Deficiency of several vitamins/minerals has been associated with higher EC incidence, with the most pronounced effect observed in the developing countries [64]. Previous studies in Iran have reported high rates of vitamin/mineral deficiencies among EC patients [42-44]; which is in line with previous research showing deficiency of zinc [113], calcium [114] and potassium [97] to be widespread among EC patients. Calcium intake from foods in this study was associated with about 50% reduction in ESCC risk. However, supplemental calcium intake has previously failed to show beneficial effects on EC risk, which could been explained by the confounding effect of higher calcium supplement intakes in the form of GERD medication by cases compared to the controls [67].

This study has several limitations. Firstly, as with other case-control studies, recall bias and selection bias were inevitable. In case-control studies, there is the possibility that cases may recall their diets differently after a cancer diagnosis. However, our participants were generally of low literacy and socioeconomic status with little knowledge about the role of diet and nutrients in the cancer risk, which should have reduced the possibility of recall bias. Moreover, using hospital controls and administering validated FFQs by trained interviewers in a hospital setting might have further reduced the recall bias and improved comparability of information of cases and controls [115,116]. With regards to the selection bias, high participation rates (94% among cases and 91% among controls) in this study minimized the potential for selective participation according to the lifestyle practices (such as diet).

Not having data on participants' alcohol consumption was yet another barrier. Our subjects refrained from reporting their alcohol intake due to the fact that consuming alcohol is legally prohibited in Iran [117,118]. In addition, since the import of alcoholic beverages is banned in Iran, the contents of alcoholic beverages that Iranians consume may differ from those consumed in other countries [117,118]. On the other hand, the Iranian FCT does not provide data on any type of alcoholic beverages [54]. Opium use was also not answered by our participants due to the cultural barriers and sensitivity of this issue among Iranian population [10], which could have resulted in confounded estimates in the present study due to the possible role of opium in ESCC risk. However, it has been suggested that opium contributes to ESCC development only in a subgroup of patients and not in the majority [6,19]. In contrast to low-incidence regions for EC, a much smaller proportion of esophageal cancer cases are attributed to alcohol, tobacco and opium use in high-risk regions [9,13,19,119]. This suggests a more prominent role for nutritional deficiencies in EC development in high-risk areas, such as Iran and China, where a larger number of cases could be attributed to insufficient nutritional intakes [19,28,42,44,75,102,120].

The third limitation is the possibility of some micronutrient misclassification due to not having data on supplement use. However, it has been suggested that dietary supplements and fortified foods, mask the beneficial effect of food intakes in reducing the cancer risk [121] and some of them have independent positive association with esophageal cancer risk [85]. In addition, supplement intake is very uncommon among our population since the majority of participants in the present study were rural dwellers with little or no education and low socioeconomic status. The average monthly family income in Iran is about 975 US$ [122] while in the present study only one of the controls had an income higher than this average and there were no significant differences between cases and controls (160.91 US $ in cases and 189.69 US $ in controls). This might relate to the sampling method in the present research which was performed in general hospitals of one of the high-risk regions of Iran. Generally, lack of information on supplement use would most likely have negligible effects on our estimates and if anything, would likely results in underestimation of association between ESCC and nutrient intakes.

Another limitation of the present study was using a semi-quantitative FFQ, which despite its common use for characterizing the habitual dietary intakes, is well-recognized for its weakness in quantification of nutrient intakes [123]. Using a semi-quantitative dietary assessment tool limits our conclusions mostly to comparisons between cases and controls and hence conclusions about adequacy of diet are relative and should be interpreted by caution, since these types of comparisons generally overestimate the true effect of exposure on the outcome. However, the theoretical basics that formed the food frequency method have been based on the good correlation of "frequency" of food intake and the "total weights" of the same foods consumed over a several-day period [124,125]. However, the potential source of error in the use of FFQ in the present study results from lack of a standardized Iranian FCT [118], although we employed the same FCTs used for validation of the Iranian FFQ [51,52]. The fundamental concept behind the calculation of nutrients from FCTs is that the nutrient contents of specific foods are relatively constant, and their variability might not be large enough to distort calculations [126]. In addition, much of the errors relating to sample-to-sample variation in nutrient composition of foods are reduced by using the estimates of long-term nutrient intakes obtained from the FFQs [126].

Another drawback of this study was using nutrient values in the statistical analyses without directly referring to the foods which contributed most to the nutrient intake and its variation. According to Willet, an optimal approach to epidemiologic analysis is to employ both foods and nutrients to represent diets [127]; in this way the case for causality is strengthened when an association is observed both with the overall intake of a nutrient and also with more than one source of that nutrient, especially when the food sources are different. Previously we evaluated the role of food group intakes in the etiology of esophageal squamous cell carcinoma (ESCC) among the same population [50]; by conducting the present study, we aimed at providing preliminary evidence on the extent to which certain nutrients could influence the ESCC risk in such a high-risk region.

Another potential source of error is that several naturally continuous variables (e.g. BMI, physical activity) were categorized for the purpose of analysis, which might have increased the possibility of residual confounding and decreased the precision and power of the study. However, we compensated for this limitation by choosing categories with multiple sufficiently narrow intervals to decrease the residual confounding and heterogeneity of subjects within intervals.

Additionally, availability of B-vitamins is influenced by diet, supplement use, alcohol consumption and generic polymorphism and B-vitamins are all involved in one-carbon metabolism which requires vitamin B_2_, B_6_, B_9 _and B_12_. This complexity added to the problems of using estimated intake of nutrients obtained at one point in time must be considered when interpreting the results of this study. Small sample size is also a limitation which might have resulted in unstable results and extreme relative risk estimates observed in some of the subgroups, although this is one of the largest sample series in an Iranian population and a number of strong consistent findings have emerged from this sample [45,50].

This study has several strengths. Firstly, eliminating information bias associated with use of proxy data enabled us to consider numerous potential cofounders. Detailed assessment and adjustment for several important confounders and total energy intake are other important aspects of this study. We attempted to reduce the measurement error and false-positive effect by calculating nutrient intake residuals standardized for total energy intake rather than reporting absolute nutrient values. This further accounted for the confounding effect of total energy intake on nutrient intake estimates [127].

In the present study, a validated FFQ was used which provided subjects with the option of answering in terms of day, week or month which enhanced reporting precision considering the fact that frequency of consumption is a truly continuous variable [128]. In addition, we asked incident ESCC patients diagnosed within 6 month of the interview to recall their diets from 1 year before diagnosis in order to capture a full cycle of seasons so that responses should not be dependent on the time of the year and be representative of habitual long-term intakes [127]. This is of note since short FFQs that have been previously used for collecting dietary data are considered the main reasons for the contradictory findings on the role of nutrients in cancer risk [56]. In addition, 24-hour dietary recalls which have been used in several studies to assess recent or current diets in relation to cancer risk, could severely compromise the accuracy of mean intake estimates due to the day-to-day variation in dietary intakes [129]. Moreover, study design further limits the reliability of short-term recalls in case control studies, since dietary recall provides information on post-diagnosis diet, while the relevant exposures have occurred earlier [127]. Given the long latency period of cancer, remote dietary intakes are far more important than the recent diet in cancer incidence studies since current dietary intake underestimates the true role of diet in cancer etiology [130].

Finally, evaluating the nutrient-cancer relationship in a population without mandatory nutrient fortification, where supplement use is uncommon and nutrient intakes are low has enabled us to capture the association of nutrients and ESCC more clearly compared to previous studies [22]; as such, results of the present study should be considered representative of the influence of vitamins and minerals that are found naturally in foods.

## Conclusion

In conclusion, findings of the present study suggest that higher SFA, cholesterol, discretionary calorie, sodium and fat intakes significantly increase the risk of ESCC, whereas dietary antioxidants, especially folate, vitamin E and selenium could prevent the damage to the esophagus caused by oxidative stress, even if consumed in moderate amounts. Our results suggest that dietary patterns rich in carbohydrate, dietary fiber and (n-3) PUFAs along with high physical activity and low consumption of hot foods and beverages and fried/barbecued meals should be promoted in order to prevent ESCC in Iranian population. However, prospective cohort studies that evaluate diet before the cancer diagnosis, as well as interventional studies that address nutrient deficiencies are warranted to clarify whether changes in dietary practices and/or vitamin and mineral supplementation can reduce the incidence of ESCC in Iran.

Overall, this study has mainly the character of a pilot hypothesis-generating study conducted in a region with low prevalence of alcohol consumption and high rates of ESCC, allowing a further search for risk factors of this cancer site. It is recommended that these findings should be replicated, particularly in comparable samples (i.e., those who have low alcohol and supplement use) to identify dietary factors that could substitute for the dominating role of alcohol and smoking for cancers of the upper gastrointestinal tract seen in many Western societies.

## Competing interests

The authors declare that they have no competing interests.

## Authors' contributions

M.J contributed to the conception and design of this study, data analysis, interpretation and drafting of the manuscript. B.R assisted in conception, design, data acquisition, analysis and drafting of manuscript. B.H collected the data and participated in designing the study. M.J. critically reviewed and helped to draft the manuscript. C.G provided methodological feedback and gave the final approval to the manuscript to be published. All authors have read and approved the final manuscript.

## Supplementary Material

Additional file 1 **Calorie-adjusted mean values among esophageal cancer cases and controls, and range of macronutrient intakes in tertile categories of selected macronutrients in a case-control study in Iran**. The file contains information about the mean macronutrient intakes among cases and controls Click here for file

Additional file 2 **Calorie-adjusted mean values among esophageal cancer cases and controls, and range of micronutrient intakes in tertile categories of selected micronutrients in a case-control study in Iran**. The file contains information about the mean micronutrient intakes among cases and controls Click here for file
