# Supplementary material for: Macronutrients, vitamins and minerals intake and risk of esophageal squamous cell carcinoma: a case-control study in Iran
Source: Nutr J. 2011 Dec 20;10:137. doi: 10.1186/1475-2891-10-137 (PMC3260093; doi:10.1186/1475-2891-10-137)
Supplement: Additional file 1 — Calorie-adjusted mean values among esophageal cancer cases and controls, and range of macronutrient intakes in tertile categories of selected macronutrients in a case-control study in Iran. The file contains information about the mean macronutrient intakes among cases and controls [file 1475-2891-10-137-S1.DOC]

**Additional file 1.** Calorie-adjusted mean values among esophageal cancer cases and controls, and range of macronutrient intakes in tertile categories of selected macronutrients in a case-control study in Iran

|  | **Mean (SD)^1^** | |  | **Tertiles of Intake** | | |
| --- | --- | --- | --- | --- | --- | --- |
| **Macronutrients** | **Case** | **Control** |  | **Tertile1** | **Tertile2** | **Tertile3** |
| Total energy, *Kcal* | 2174.7 ±594.0 | 2091.0 ±485.3 |  | 816.50-1663.00 | 1675.00-2224.01 | 2229.80-3390.11 |
| Total fat, *g* | 69.18±4.51 | 68.15±2.72 |  | 20.70-54.01 | 54.57-75.87 | 75.89-171.40 |
| SFA, *g* | 32.36±1.08 | 17.28±1.34^*^ |  | 4.41-15.41 | 15.55-22.29 | 22.63-65.13 |
| PUFA*, g* | 16.53±0.65 | 18.17±1.67 |  | 4.05-13.44 | 13.64-18.88 | 18.90-67.36 |
| MUFA*, g* | 22.45±1.43 | 22.32±0.96 |  | 6.02-18.19 | 18.44-25.27 | 25.32-70.49 |
| (n-3)fatty acids*, g* | 0.92±0.02 | 1.02±0.06^*^ |  | 0.00-0.07 | 0.08-0.94 | 0.95-2.53 |
| Dietary fiber, *g* | 18.06±4.47 | 39.77±2.57^*^ |  | 3.50-13.88 | 13-94-29.52 | 30.95-44.37 |
| Carbohydrate, *g* | 249.87±9.87 | 278.64±7.45^*^ |  | 28.19-228.80 | 229.30-306.40 | 306.82-549.70 |
| Protein, *g* | 83.32±3.18 | 87.49±1.27 |  | 14.50-69.11 | 69.68-95.44 | 96.21-207.90 |
| Cholesterol, *mg* | 248.53±8.57 | 244.89±10.93 |  | 58.66-161.33 | 165.44-295.60 | 296.80-918.50 |
| Vegetable Oil, *g* | 29.98±1.27 | 34.34±1.13^*^ |  | 17.89-37.14 | 48.92-56.60 | 57.39-84.92 |
| Discretionary calorie, *% total energy intake* | 65.27±3.62 | 50.12±1.28^*^ |  | 12.20-35.06 | 36.93-54.29 | 57.64-68.13 |

SFA: Saturated fatty acid; PUFA: Poly unsaturated fatty acid; MUFA: Mono unsaturated fatty acid

^*^Statistically significant mean difference between cases and controls (p<0.05)

^1^Means are adjusted for total energy intake using analysis of covariance
