# Supplementary material for: Macronutrients, vitamins and minerals intake and risk of esophageal squamous cell carcinoma: a case-control study in Iran
Source: Nutr J. 2011 Dec 20;10:137. doi: 10.1186/1475-2891-10-137 (PMC3260093; doi:10.1186/1475-2891-10-137)
Supplement: Additional file 2 — Calorie-adjusted mean values among esophageal cancer cases and controls, and range of micronutrient intakes in tertile categories of selected micronutrients in a case-control study in Iran. The file contains information about the mean micronutrient intakes among cases and controls [file 1475-2891-10-137-S2.DOC]

**Additional file 2.** Calorie-adjusted mean values among esophageal cancer cases and controls, and range of micronutrient intakes in tertile categories of selected micronutrients in a case-control study in Iran

|  | **Mean (SD)^1^** | |  | **Tertiles of Intake** | | |
| --- | --- | --- | --- | --- | --- | --- |
| **Macronutrients** | **Case** | **Control** |  | **Tertile1** | **Tertile2** | **Tertile3** |
| Vitamin A, *RAE* | 423.75±11.29 | 535.91±9.30^*^ |  | 63.90-461.40 | 498.24-1403.00 | 1471.23-3828.03 |
| β-carotene*,µg* | 326.94±14.27 | 1792.47±15.53^*^ |  | 15.66-482.72 | 488.40-1319.38 | 1429.58-5122.46 |
| Vitamin D, *µg* | 1.08±0.13 | 1.47±0.10^*^ |  | 0.00-0.75 | 0.77-1.42 | 1.44-5.58 |
| Vitamin E, *mg TE* | 4.21±1.20 | 8.01±0.42^*^ |  | 0.61-2.40 | 2.45-7.54 | 7.80-30.91 |
| α-tocopherol*,mg* | 6.87±0.36 | 13.66±1.35^*^ |  | 0.80-5.05 | 5.08-9.17 | 9.43-32.61 |
| Thiamine, *mg* | 1.03±0.09 | 1.20±0.02^*^ |  | 0.24-1.59 | 1.62-1.97 | 1.99-6.96 |
| Riboflavin, *mg* | 1.07±0.09 | 1.25±0.05^*^ |  | 0.42-1.11 | 1.12-2.30 | 2.34-4.47 |
| Niacin, *mg* | 12.80±0.99 | 15.70±0.43 |  | 2.39-5.97 | 15.10-23.85 | 27.20-46.61 |
| Panthothenic acid, *mg* | 4.45±0.93 | 4.46±0.09 |  | 0.90-3.45 | 3.47-4.94 | 4.96-19.84 |
| Vitamin B6, *mg* | 1.15±0.19 | 1.71±0.03^*^ |  | 0.22-1.22 | 1.23-1.96 | 1.97-11.48 |
| Folate, *µg* | 233.96±24.38 | 379.15±12.01^*^ |  | 43.14-172.00 | 172.90-406.49 | 426.21-689.0 |
| Vitamin B_12_, *µg* | 2.10±1.53 | 3.76±0.58^*^ |  | 0.49-2.41 | 2.51-2.69 | 3.33-11.91 |
| Vitamin C, *mg* | 83.99±4.25 | 98.59±16.11^*^ |  | 2.45-61.10 | 61.24-80.10 | 83.41-217.72 |
| Iron*, mg* | 9.34±1.02 | 13.03±0.95^*^ |  | 1.51-9.18 | 10.19-15.67 | 18.16-31.35 |
| Calcium*, mg* | 843.98±22.20 | 1142.62±34.50^*^ |  | 208.30-786.50 | 792.10-924.00 | 1131.13-2545.50 |
| Phosphorous*, mg* | 953.17±32.53 | 1175.05±29.54^*^ |  | 211.00-835.04 | 848.60-1063.31 | 1205.34-3082.01 |
| Potassium, *mg* | 3120.52±34.69 | 3152.72±35.83 |  | 518.60-1991.50 | 2110.88-3024.77 | 3040.10-5349.40 |
| Sodium, *mg* | 4022.53±31.60 | 3451.54±16.66^*^ |  | 986.60-2116.40 | 2171.00-3748.57 | 3819.79-11542.00 |
| Zinc*, mg* | 8.90±0.91 | 10.84±0.23 |  | 1.89-7.32 | 7.38-10.44 | 11.48-24.82 |
| Methionine, *g* | 1.06±0.69 | 1.75±0.98^*^ |  | 0.36-0.98 | 1.08-1.49 | 1.50-6.47 |
| Selenium, *µg* | 0.12±0.02 | 74.82±4.59^*^ |  | 0.02-0.09 | 0.19-85.15 | 89.18-90.14 |

RAE=Retinol Activity Equivalents; TE= Tocopherol Equivalents

^*^Statistically significant mean difference between cases and controls (p<0.05)

^1^Means are adjusted for total energy intake using analysis of covariance
